# The impact of alkaline treatments on elasticity in spruce tonewood

**DOI:** 10.1038/s41598-022-17596-z

**Published:** 2022-08-03

**Authors:** Raffaele Malvermi, Michela Albano, Sebastian Gonzalez, Giacomo Fiocco, Fabio Antonacci, Marco Malagodi, Augusto Sarti

**Affiliations:** 1 Musical Acoustics Lab at the Violin Museum of Cremona, DEIB-Politecnico di Milano, Cremona Campus, Cremona, Italy; 2 Arvedi Laboratory of non-Invasive Diagnostics, CISRiC, University of Pavia, Via Bell’Aspa 3, 26100 Cremona, Italy; 3 Department of Musicology and Cultural Heritage, University of Pavia, 26100 Cremona, Italy

**Keywords:** Mechanical engineering, Acoustics, Mechanical properties

## Abstract

It is commonly believed that violins sound differently when finished. However, if the role of varnishes on the vibrational properties of these musical instruments is well-established, how the first components of the complete wood finish impact on the final result is still unclear. According to tradition, the priming process consists of two distinct stages, called *pre-treatment* and *sizing*. The literature reports some recipes used by old Cremonese luthiers as primers, mainly based on alkaline aqueous solutions and protein-based glues. In this manuscript, we analyze the impact of these treatments on the mechanical properties of the material. The combination of two pre-treatments and three sizes is considered on nine different plates. We compare the vibrational properties before and after the application and assess the effects of the different primers, also supported by finite element modeling. The main outcome is that the combination of particular treatments on the violin surface before varnishing leads to changes not only to the wood appearance, but also to its vibrational properties. Indeed pre-treatments, often considered negligible in terms of vibrational changes, enhance the penetration of the size into the wood structure and strengthen the impact of the latter on the final rigidity of the material along the longitudinal and radial directions.

## Introduction

Wood finish is one of the most fascinating and delicate processes in violin making. It is a common belief, in fact, that different aspects of the violin, from its durability to its visual appeal and perceived timbre, depend on how the maker designs the finish and applies it to the instrument. According to Koen Padding^1^, it is the finish what makes or breaks a violin that sounds very good in ‘white’. In this regard, classical Cremonese varnishes set the bar in aesthetic terms thanks to the transparent yet colorful effect that is still appreciated by musicians nowadays. Following the tradition, luthiers still pay great attention in refining their recipes to reach a similar trade-off between elegant appearance and desired sound radiation capability.

Because of the important role that this step has in the uniqueness of a violin, makers still keep their own methods and discoveries secret. Nonetheless, some common practices can be outlined. The surface of the tonewood usually undergoes three main types of treatment during the finishing process. First, the plates are pre-treated with alkaline solutions to enhance wood durability and prevent biological attacks^2^. We will refer to these as *pre-treatments*. In a second step, the wood is prepared for varnish through the application of a protein-based compound that generally includes animal glues or caseinate. These glues, denser than the previous solutions, can seal the surface pores and avoid a potential soaking of the varnish. The process is known under different names: sizing, sealing, filling or grounding. In this manuscript, we will denote it as *sizing*. After these *priming* steps, the violin is ready to be varnished.

The use of primers in the past has been proved by the analytical investigation carried out on historical artworks^3–6^ and musical instruments^7^, and its impact has been mainly observed in prevention and wood appearance^8,9^, i.e. leading to either a darkening or discolouring of the material. Among the physical and chemical pre-treatments available in the art and crafts literature, treating the wood in alkaline medium is one of the most reported. In particular, two main pre-treatments can be considered: on the one hand the ammonia fuming, which simulates the old common practice to place the violin on a smoldering dung pit^10^ and, on the other hand, a lye-based solution usually applied with a brush or a sponge.

To the best of our knowledge, research has focused the attention only on varnish, probably driven by the greater amount of information available on its chemical composition with respect to primers^11,12^. Indeed, it is commonly accepted that varnish does not only contribute to the aesthetic appearance of violins, but it also plays an important role in the wood protection^13^ and sound production^14–20^. Conversely, very few works suggested possible chemical compositions for primers and provided a tentative explanation about their function^2,21^. Primers may produce a variation on the wood structure extended in depth to a relevant portion of its thickness. However, how deep a prescribed combination of pre-treatment and size can penetrate inside the tonewood and which changes the primer induces to the elastic properties of the treated material are still open questions that call for an in-depth study from the standpoint of vibrational analysis.

By using a multi-disciplinary approach including vibration tests, Finite Element (FE) modeling and Scanning Electron Microscopy with Energy Dispersive X-ray (SEM-EDX) analysis, we studied the penetration of primers in the wood structure and their impact on its material properties. We first applied a selection of two alkaline pre-treatments and three protein-based sizes over one side of a set of Spruce plates, as traditionally done by luthiers which varnish only the outer surface of the violin. Vibration tests were first performed on untreated plates, and then repeated after both pre-treatment and size were applied. The data obtained in terms of modal frequencies and density allowed us to estimate the variation in the specific stiffness characterizing the treated plates with respect to the original ones. Subsequently, we employed FE analysis to fit the measurements, study the penetration depth of each primer and estimate the change in density in the portion of wood affected by treatments. Due to the small value chosen for the thickness of the specimens, and the different moisture exchange rate observed for the two faces and caused by one-sided applications, the so-called ‘flying wood’ or ‘cupping’ phenomenon occurred^22^. The consequent geometrical changes were taken into account inside the FE model. For a subset of the specimens, considerations extracted from simulations were supported with the actual evaluation of the penetration depths through SEM-EDX.

**Figure 1:**
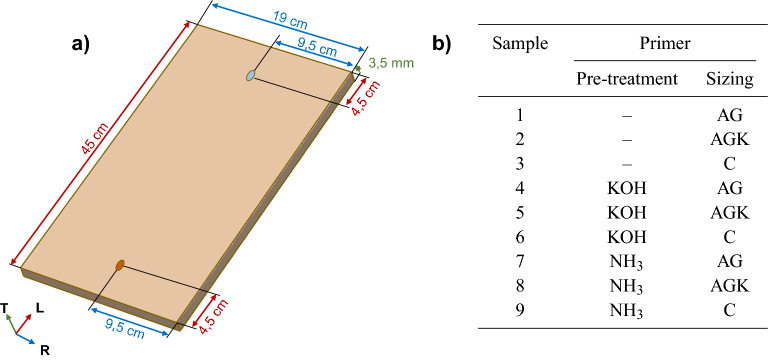
(**a**) Diagram of the plate geometry, with the location of the excitation (highlighted in orange) and measurement ( highlighted in light blue) points chosen for hammer impact testing. (**b**) Table listing the nine Spruce plates and details on the different primers applied.

## Results

**Figure 2:**
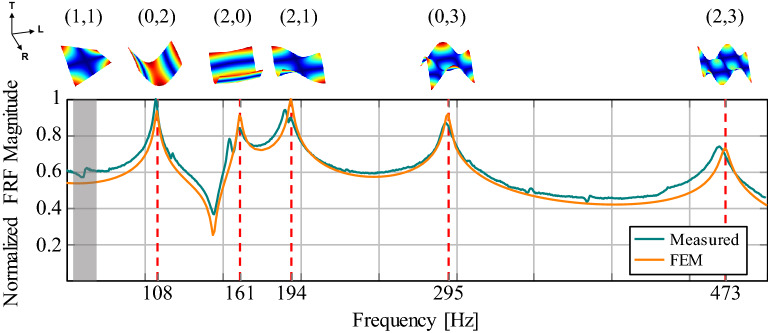
Comparison between FRF measured (in cyan) and FEM simulation (in orange) obtained with the estimated elastic constants. In the example, plate 4 is considered. The resulting magnitudes are normalized between 0 and 1. Measurements from white specimens were fitted as in^23^ and using Rayleigh damping with constants $\alpha =10$ [${\hbox {s}^{-1}}$] and $\beta =2\times 10^{-6}$ [s], according to^24^. The eigenfrequencies obtained numerically and corresponding to resonances in the measure are highlighted with dashed red lines. In the measurement points chosen, modes with a nodal line along the longitudinal axis of symmetry are attenuated, e.g. (1,1) (colored in gray), making the peaks of interest easier to identify. The mode shape associated to each resonance is depicted above the plot, denoted with a notation based on nodal lines and widely used in the literature^25^.

Frequency Response Functions (FRFs) were collected on a pair of fixed points over the specimens (Fig. 1a) to study how the treatments affect the free vibration and the stiffness of tonewood. Different chemicals were combined and applied to a total of six plates (Fig. 1b): (i) an alkaline solution based on potassium (KOH); (ii) an alkaline medium based on ammonia ($\hbox {NH}_{3}$); (iii) animal glue (AG); (iv) animal glue with the addition of kaolin (AGK); (v) a sizing based on caseinate (C). Three additional plates were only sized. We estimated the initial elastic parameters of the untreated samples, which we will refer to as *white*, as in^23^. Figure 2 shows the comparison between the FRFs measured for plate 4 and the result of a FEM simulation using the estimated material properties.

**Figure 3:**
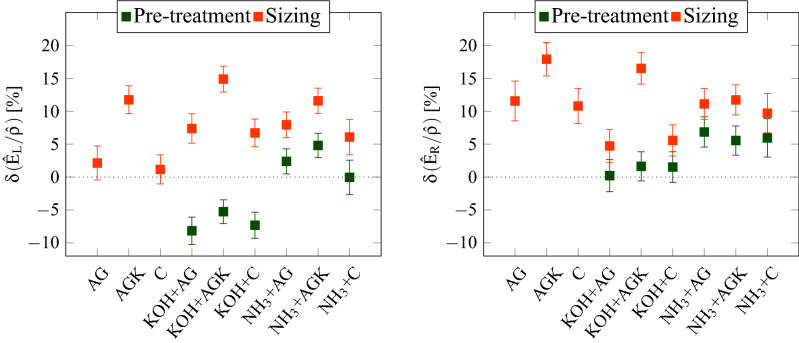
Relative variation in the longitudinal and radial specific stiffness observed at the end of each treatment stage (green: pre-treatment, orange: sizing). Values are expressed in percentage and represented with squares. Error bars represent the estimation error. Left: Estimated variation for the longitudinal specific stiffness $\delta (\hat{E}_L/\hat{\rho })$, with a maximum error of 2.7%; Right: Estimated variation for the radial specific stiffness $\delta (\hat{E}_R/\hat{\rho })$, with a maximum error of 3.0%. Estimations are based on Caldersmith’s equations^26^, assuming the contribution of Poisson’s ratios as negligible.

### Influence on wood stiffness

The impact of pre-treatments and sizing on wood stiffness was assessed by measuring the change in the longitudinal and radial Young’s moduli and in the density of the specimens. We focused on these material properties since they have been proved to be important for determining the vibroacoustic properties of complete instruments^23,27–29^. Indeed, their correlation with the first modes of the instrument is found to be higher than others^30^. Moreover, luthiers usually focus on the Young’s modulus along the principal dimensions of the wedge as they can infer them empirically^31^ and they are used to define acoustic indicators for instruments (e.g. anisotropy ratio^15^).

The Young’s moduli were estimated from the frequency of modes (0,2) and (2,0) using the Caldersmith’s formula^26^. The relative change in the specific stiffness, i.e. the ratio between the measured Young’s modulus $\hat{E}$ and the measured density $\hat{\rho }$, was computed along the longitudinal and radial wood directions after each treatment stage. The corresponding material properties obtained for the white samples where used as reference in the relative difference.

Figure 3 shows the resulting values of $\delta (\hat{E}_L/\hat{\rho })_{t}^j$ and $\delta (\hat{E}_R/\hat{\rho })_{t}^j$, estimated for each plate *j* after the application of each treatment *t*. Green markers are associated to values obtained after the pre-treatment ($t=p$) while orange markers correspond to values after the sizing ($t=s$). Error bars represent the estimate error, computed starting from the uncertainties in the measured FRF, the plate dimensions and the density.

The two proposed pre-treatments show opposite behaviors with respect to wood stiffness. On the one hand, specimens pre-treated with KOH (plates 4–6) are characterized by an average decrease equal to − 6.9(±2.0)% in $\delta (\hat{E}_L/\hat{\rho })_{p}^j$ while the estimated $\delta (\hat{E}_R/\hat{\rho })_{p}^j$ is comparable to the measurement error. On the other hand, plates pre-treated with $\hbox {NH}_{3}$ (7–9) show an unclear modification of the along-grain stiffness, i.e. average $\delta (\hat{E}_L/\hat{\rho })_{p}^j$ equal to 2.4(± 2.2)%, while an average increase of 6.1$(\pm$ 2.5)% can be noticed in $\delta (\hat{E}_R/\hat{\rho })_{p}^j$. However, the pre-treatment alone leads to variations that are limited to 10%. The same variation can be observed within wood blocks cut from the same tree^32^.

Changes in the specific stiffness become relevant only when plates are also sized. Indeed, $\delta (\hat{E}_L/\hat{\rho })_{s}^j$ is greater than 10% if the treatment combination AGK (plates 2, 5 and 8) is used. If pre-treatments analysed in combination with AG or C (plates 4, 6, 7 and 9) are considered, $\delta (\hat{E}_L/\hat{\rho })_{s}^j$ is in average 7.0$(\pm$2.3)%. Conversely, the application of glues without additional particles (AG, C) and without a pre-treatment shows an increase in line with the measurement error (plates 1 and 3). The radial specific stiffness seems to be affected by every sizing, regardless of the presence of pre-treatments. Indeed, $\delta (\hat{E}_R/\hat{\rho })_{s}^j$ is always larger than 7%, also for plates which underwent no pre-treatment (1-3). Moreover, the use of animal glue with a dispersion of kaolin (AGK) leads to a more pronounced stiffening of the wood structure across the grains, with an increase up to 20%. Interestingly enough, primers based on KOH and organic glues (KOH+AG, KOH+C) seem to impact moderately on $\delta (\hat{E}_R/\hat{\rho })_{s}^j$, with variations around 5%.

**Figure 4:**
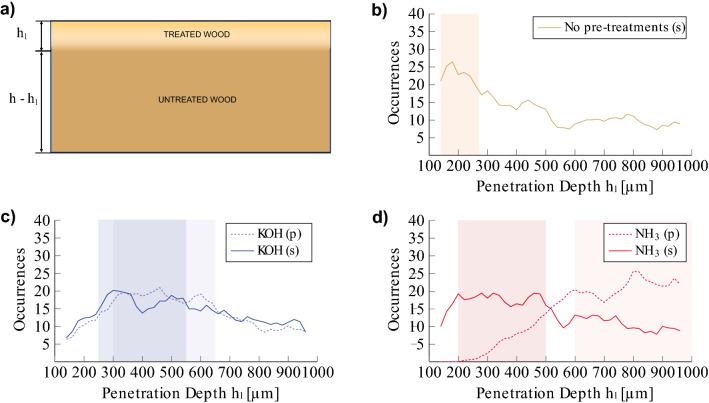
(**a**) Diagram of the transversal profile assumed in the FE model for the characterization of the treated side of the specimen. (**b**) Distribution of penetration depths $h_l$ making the FE model best approximate the modal frequencies of plates 1-3, only sized. (**c**) Distribution of penetration depths $h_l$ making the FE model best approximate the modal frequencies of plates 4-6, pre-treated with KOH. (**d**) Distribution of penetration depths $h_l$ making the FE model best approximate the modal frequencies in plates 7-9, pre-treated with $\hbox {NH}_{3}$. Histograms are evaluated when plates are only pre-treated ($t=p$, dashed lines) and after the application of the complete primers ($t=s$, solid lines). Colored regions highlight the ranges of preferential values characterizing each combination of treatments.

### Treatment penetration

In order to analyze the penetration of the treatments, we employed the FEM-based measurement fitting explained in^23^, modifying the FE model. The model was defined assuming a change in the parameters of the treated side of the plate with respect to untreated wood, together with the introduction of the arching due to the cupping phenomenon. Figure 4a shows the transversal profile of the ’virtual’ specimen, in which the plate consists of two different materials. The material on top, which is assumed to be noticeably thinner than the other, models the penetration of the treatment.

We simulated the vibrational behavior of the virtual plate varying the penetration depth $h_l$ randomly in the range [100, 1000] $\upmu \hbox {m}$ and the most relevant properties of the material on top (i.e. density, longitudinal and radial Young’s moduli, longitudinal to radial shear modulus) around the corresponding optimal values found for the white plate. The properties of the second material were left fixed during the simulation campaign, and equal to those estimated for the untreated wood.

With the new model, the optimization performed did not provide a unique set of optimal parameters for the treated wood section, since the cost function used was severely affected by local minima. Therefore, we analyzed the histogram of the penetration as obtained from the local minima of the cost function.

The analysis revealed that a range of ‘preferential’ depths exists for each primer under study. Figure 4 shows the distribution of input depths $h_l$ that, given a specific treatment, make the FE model best approximate the modal frequencies measured in the corresponding plate. Distributions are represented with different smoothed histograms for the pre-treatment ($t=p$, dashed lines) and the sizing ($t=s$, solid lines) stages, and grouped by pre-treatment used: plates 1–3 (only sized, Fig. 4b), plates 4–6 (pre-treated with KOH, Fig. 4c) and plates 7–9 (pre-treated with $\hbox {NH}_{3}$, Fig. 4d). For each histogram, the range with the largest number of occurrences (i.e. for which the number of occurrences is greater than 80% of the max) is highlighted in color.

If we look at the penetration depth of the pre-treatments (dashed lines), a range of depths concentrated within 300–650 $\upmu \hbox {m}$ for KOH (plates 4–6, highlighted in light blue) and between 600 and 900 $\upmu \hbox {m}$ for $\hbox {NH}_{3}$ (plates 7-9, highlighted in light red) are observed.

After the application of the sizing, these ranges shift to lower values. The histogram obtained for the KOH+AG, KOH+AGK and KOH+C primers (plates 4–6, solid blue line) shows a slight shift of the region with the largest number of occurrences from 300–650 $\upmu \hbox {m}$ to 250–550 $\upmu \hbox {m}$ (colored in blue). The effect of sizes seems more prominent for glues combined with $\hbox {NH}_{3}$ (plates 7–9, solid red line) where the new range is located between 200 and 500 $\upmu \hbox {m}$ (colored in red).

Plates where sizes were applied directly show small values of $h_l$, concentrated between 140 and 270 $\upmu \hbox {m}$ (highlighted in yellow).

**Figure 5:**
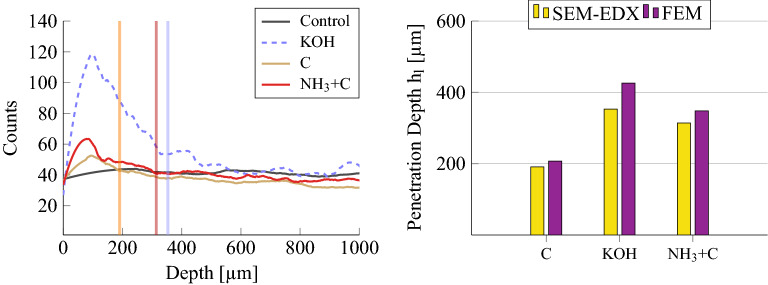
Left: Semi-quantitative line profiling of potassium (K) analyzed with SEM-EDX on plates subject to potassium-based treatments (i.e. involving KOH and C). The lines represent the potassium concentration evaluated at different depths while colored regions highlight the feasible limits of interaction between the wood and the different treatments. The limits were estimated by fitting a bilinear model to each profile decay; Right: Comparison between the penetration depths estimated from SEM-EDX profiles (yellow bars) and the mean values of the preferential depth ranges found through FE analysis (violet bars). The analysis is limited to plates subject to potassium-based treatments.

Similar considerations on the penetration depth of primers can be formulated by observing the results of the SEM-EDX transversal analysis on cross-sectional samples, which reveals the variation of the potassium (K) relative abundance as a function of depth.

Figure 5 (Left) shows the profiles measured for the plates treated with KOH, C and $\hbox {NH}_{3}$+C, along with a control plate, left untreated. Indeed, even though K was detected at lower counts also in the wooden structure, the EDX analysis highlighted that K can be considered as a marker of the potassium-based treatments (i.e. involving KOH and C).

It can be seen that when a section of the sample has been treated, the profile shows higher counts down to a certain depth. The profile becomes then comparable with the control for higher depths. We thus interpreted the change in the slope of the K counting profile as the limit of interaction between the portion of wood which probably underwent chemical modification or impregnation and the rest of the sample.

The limits of interaction were thus estimated by fitting the decaying part of the profiles with a piece-wise linear (or bilinear) function. The results are shown with vertical lines in Fig. 5 (Left) with the corresponding profile color.

Figure 5 (Right) shows a final comparison between the penetration depths extracted from the SEM-EDX measurements (yellow bars) and the mean of the range of preferential depths found for plates 3, 6 and 9 through FE analysis (violet bars). On the one hand, the potassium counting profiles reveal larger counts until 200 $\upmu \hbox {m}$ for C (solid brown line), around 350 $\upmu \hbox {m}$ for KOH (dashed blue line) and around 300 $\upmu \hbox {m}$ for $\hbox {NH}_{3}$+C (solid red line). On the other hand, an average preferential depth of 191 $\upmu \hbox {m}$, 426 $\mu \hbox {m}$ and 348 $\upmu \hbox {m}$ was found for C, KOH and $\hbox {NH}_{3}$+C, respectively.

**Figure 6:**
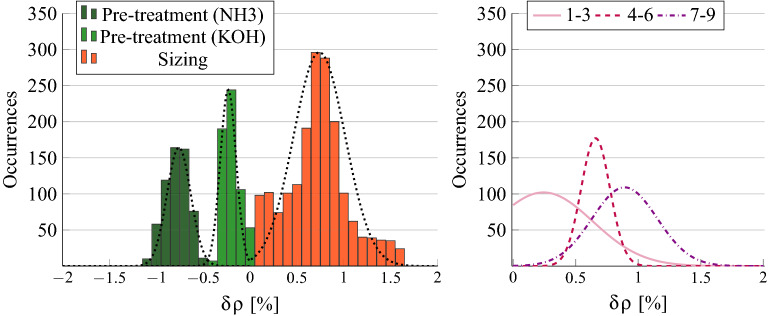
Left: Distribution of changes in the equivalent density of the FE model $\delta \rho$. The equivalent density is obtained as the weighted sum of the estimated layer density $\rho _l$ and the density measured originally. The weights used are the penetration depth $h_l$ and $h-h_l$, respectively. Histograms are computed after both the treatment stages (pre-treatment: green, sizing: orange) collecting the values in percentage such that the FE model best approximate the modal frequencies in each plate. Dashed lines correspond to the histogram fits by means of Gaussian probability density functions; Right: Gaussian fit for $\delta \rho$ after the sizing stage, computed for plates grouped by pre-treatment used. Pre-treatments induce a density increase due to the sizing which is greater than the variation encountered if plates are only sized.

### Density change in the treated section

We performed the analysis conducted on the distribution of the simulated penetration depths also on the other control parameters characterizing the material on top of the FE model. The resulting distributions revealed different material changes as a function of the primer.

Figure 6 (Left) shows the histograms of the change in the equivalent density of virtual specimens. The equivalent density is obtained as the weighted sum of the estimated layer density $\rho _l$ and the original density (i.e. measured for the white plates). The weights used are the penetration depth $h_l$ and $h-h_l$, respectively. The variations are collected from all the plates after each treatment stage (pre-treatment: green bars, sizing: orange bars) and expressed in percentage. A Gaussian fit was performed for each histogram to estimate the mean $\mu$ and the standard deviation $\sigma$. Figure 6 shows the resulting probability density functions (dashed black lines) superimposed to the corresponding histograms. The parameters of the fit obtained for $\hbox {NH}_{3}$ only are ($\mu _{NH_3}^p=-0.78\%$, $\sigma _{NH_3}^p=0.14\%$), the ones obtained for KOH only are ($\mu _{KOH}^p=-0.22\%$, $\sigma _{KOH}^p=0.09\%$) and those obtained after the sizing (regardless of the pre-treatment used) are ($\mu _{ALL}^s=0.72\%$, $\sigma _{ALL}^s=0.29\%$). It can thus be noticed that plates affected by alkaline pre-treatments show a reduced density, which is lower when $\hbox {NH}_{3}$ is used, while the application of a denser matter such as protein-based glues results in an average increase with respect to the original value.

By analyzing more in detail the variation induced after the sizing, it can be seen that pre-treatments induce a density increase which is greater than the variation encountered if plates are only sized. Figure 6 (Right) shows the Gaussian fit of the histograms computed considering the application of the complete primers and grouping the plates by the pre-treatment used. In this case we obtained ($\upmu _{U}^s=0.30\%$, $\sigma _{U}^s=0.42\%$) for the untreated (U) plates 1–3 (solid pink line), ($\upmu _{KOH}^s=0.67\%$, $\sigma _{KOH}^s=0.13\%$) for plates 4–6 (dashed purple line) and ($\upmu _{NH_3}^s=0.94\%$, $\sigma _{NH_3}^s=0.26\%$) for plates 7–9 (dashdotted line). Interestingly enough, the mean obtained for plates which underwent no pre-treatment (0.3) is lower than the ones obtained from pre-treated plates (0.67, 0.94).

The density changes obtained through FEM modeling are in line with the density values measured after each treatment stage. Indeed, an average variation of $-0.78\pm 0.12\%$ was observed after pre-treating the specimens with $\hbox {NH}_{3}$, while an average decrease equal to $-0.19\pm 0.08\%$ was found for plates treated with KOH. Concerning the plates after the sizing stage, an increase in the density was measured for both plates only sized ($0.46\pm 0.36\%$), plates pre-treated with KOH ($0.80\pm 0.21\%$) and plates pre-treated with $\hbox {NH}_{3}$ ($0.67\pm 0.10\%$).

## Discussion

In this study, a multi-disciplinary approach consisting of vibrational measurements, FEM modeling and SEM-EDX investigation has been employed to characterize the impact of Cremonese traditional alkaline pre-treatments and sizing on the elasticity and vibration of Spruce tonewood, typically used for the production of soundboards in violin making. To the best of our knowledge, this is the first systematic study in this direction. Pre-treating the wood eases the penetration of sizes into the wood pores, inducing a variation in the material parameters, such as its density and stiffness, which is greater than the one observed for wood which underwent only the sizing stage. As a consequence, also the vibrational properties of the tonewood plate are more affected. These findings may be crucial for makers, so that the contribution of each stage of the finishing to the final sound is taken into account. Indeed, the impact of the first wood treatments has been always considered marginal with respect to the varnish, and less efforts were devoted to clarify this aspect from a quantitative point of view.

Vibration tests revealed a change in the first modal frequencies of the specimens, and thus in the specific stiffness along the most relevant directions. Although the application of the pre-treatment may not induce relevant modifications by per se, the combined effect with sizes leads to an enhanced stiffening of the whole plate, especially along the wood grain. The effect is more pronounced if kaolin particles are dispersed into the treatment: as an example, the mixture of animal glue and kaolin showed the largest variation in the specific stiffness in both longitudinal and radial directions of the plate. This evidence opens the door to further investigations for characterizing the variety of inorganic particles used in wood finishes^33,34^, to study whether the particle size or concentration can control these material changes. Moreover, focusing on one treatment combination and increasing the number of specimens used may lead to more informative and representative results.

FEM modelling and SEM-EDX analysis highlighted the different penetration of alkaline-based treatments, both before and after the sizing. Analyzing the pre-treatment only, $\hbox {NH}_{3}$ may penetrate more than KOH. The different behavior highlighted for the two pre-treatments may be a consequence of the different application procedure: indeed, ammonia fuming involves the whole volume of the specimen while water-based applications such as KOH affect only the specimen’s surface.

When the combination of pre-treatment and sizing is considered, an increase in the penetration depth can be observed if compared to the direct application of sizes on untreated plates, especially when KOH is applied. This result, together with a reduction of the density in the upper portion of pre-treated specimens, and thus of the equivalent density of specimens, may confirm the action of alkaline treatments as an artificial support in wood drying or aging^2^.

## Methods

### Materials

Nine Spruce rectangular plates (*Picea abies* L., purchased at Rivolta Wood, Italy) with average density equal to 400 ${\hbox {kgm}^{-3}}$ ($\pm 5$ ${\hbox {kgm}^{-3}}$, at 20 °C and 50% RH) and dimensions $450 \times 190 \times 3.5$ mm (L $\times$ R $\times$ T) were selected from 5 pairs of book-matched samples. The selected plates were all harvested in 2005 in Val di Fiemme, Italy, and aged in identical conditions. The specimens were stored in a humidity-controlled room at 20 °C and 50% RH during the whole measurement campaign. The moisture content of each plate has been tracked and updated after each treatment using a resistance moisture meter with surface-contact electrodes. With the same frequency of acquisition, we measured and tracked also the density. Following the finishing procedure presented in^21^, two types of alkaline medium were selected to emulate historical pre-treatments^10,33^: a 1 M aqueous solution at $pH = 13$ of potassium hydroxide (KOH - pellets, Carlo Erba, Italy) and ammonia ($\hbox {NH}_{3}$) vapor (Ammonia 30% v/v, Bresciani s.r.l. Italy). We chose ammonia fuming and potassium hydroxide as evidences of the modification of the wood structure were found on a different set of Spruce plates and a similar number of specimens per treatment combination, according to^21^. During the first stage of the campaign, KOH was spread with a brush over one face of the plates, while $\hbox {NH}_{3}$ was applied by inserting the specimens into sealed boxes, saturated with ammonia vapor, for 96 hours. At this stage, part of the samples was left untreated and kept as control. After a curing time of three months, the final measurements for the pre-treatment (*p*) stage have been accomplished. In the second stage (*s*), three different sizes have been applied with a brush: (i) a solution with 10% of rabbit glue dissolved in water (AG—rabbit glue Oporto extra, Bresciani s.r.l. Italy); (ii) a mixture of 10% rabbit glue enriched with 1% kaolin $\hbox {Al}_{2}\hbox {Si}_{2}\hbox {O}_{5}\hbox {(OH)}_{4}$ (AGK); and (iii) potassium caseinate prepared by adding casein powder (cross-linked casein, Farmacia Leggeri, Italy) to distilled water (15% w/w), subsequently dissolved in KOH solution 0.5 M (C), according to an ancient recipe^35,36^. As a result, nine different combinations of pre-treatments and sizes were studied (Fig. 1b). All the specimens underwent a further drying period of three months before the final measurements. To partially correct the cupping effect occurred in plates due to one-sided applications, weights were applied on the specimens during the drying periods.

### Vibration tests

The Frequency Response Functions (FRFs) were measured by means of hammer impact testing. A structure made of wood and rubber bands was built to simulate free boundary conditions during the test. Four rubber bands were used for the suspension of the plates, letting the sample in vertical position and minimizing the contact surface^37,38^. In this configuration, also the resting position of the hammer results vertical after the hit, avoiding the occurrence of accidental secondary strikes during the acquisition. A dynamometric hammer with light tip (086E80, by *PCB Piezotronics*) and an uniaxial accelerometer (352A12, by *PCB Piezotronics*) were used to generate an impulsive excitation and measure the harmonic response. The excitation and the measurement points were placed along the axis of symmetry parallel to the plate length, at 45 mm from the edges (i.e. 10% of the total length) as depicted in Fig. 1a. An advantage of using such pair of points on the plate is that modes less correlated to the Longitudinal and Radial Young’s moduli exhibited an attenuated peak amplitude. In this way, the peaks of interest were easily identified inside the acquired frequency responses. For each measurement, six time-domain signals of two seconds sampled at 48 kHz were acquired. FRFs were estimated following the definition of the H1 estimator^39^. Details on the estimations performed starting from measured FRFs can be found in Supplementary Information.

### FEM modeling

A two-step Monte-Carlo optimization procedure was used to characterize first the original material of the samples (i.e. before the application of any chemicals) and, at a later stage, the section of the specimens affected by the treatments. In particular, the first step consisted in the assessment of the material parameters ($\rho , E_L, E_R, E_T, G_{LR}, G_{RT}, G_{LT}, \mu _{LR}, \mu _{RT}, \mu _{LT}$) of the white plate given the corresponding set of measured modal frequencies. Since wood is an orthotropic material, a different mechanical behaviour is observed along the different axes of the (L $\times$ R $\times$ T) reference system. A Monte-Carlo sampling approach was employed to estimate the parameters. The dataset consisted of 3000 realizations of plates with varying material properties, to be used in the FE analysis. The parameters were sampled from different distributions around a nominal value. The nominal values of $E_L$ and $E_R$ were obtained through the Caldersmith’s formula from the knowledge of the modal frequencies. The remaining nominal values were taken from the literature^32^. The reference material used was Sitka Spruce, not a common material in European violin making tradition, but with mechanical properties known and similar to Spruce tonewood. A table resuming the distributions and the nominal values used for the sampling is provided in Supplementary Information (Table S1). For the FE analysis a 3D model of the specimens was fed to COMSOL Multiphysics. The resulting tetrahedron mesh was analysed multiple times with the “Solid Mechanics” module of the software, assuming free boundary conditions and defining the material of the model each time with a different tuple of the dataset. For each realization in the dataset, an eigenfrequency study was performed to compute the frequency and mode shape of the first 15 modes. The estimated parameters are those of the item in the dataset whose modal frequencies are closest to the measured ones, as in^23^. A further frequency-domain study was performed to validate the final solution, comparing the resulting FRF to the measured one. In this process, Rayleigh damping^40^ was introduced in the model with constants $\alpha =10$ $\hbox {s}^{-1}$ and $\beta =2\times 10^{-6}$ s. The damping model was chosen according to previous applications of numerical modeling in the context of musical instruments^23,24^. The initial and final values of the material parameters ($\rho , E_L, E_R, E_T, G_{LR}, G_{RT}, G_{LT}, \mu _{LR}, \mu _{RT}, \mu _{LT}$) obtained during the first step of the optimization are reported in Supplementary Information (Tables S2, S3 and S4).

The mechanical parameters of the treated layer at the treatment stages *p* and *s* were estimated through a modified version of the Monte-Carlo optimization proposed in^23^. The proposed method works under the assumption that all the plates can be modeled after pre-treatment and sizing by a two-layer structure, the upper and lower layers being, respectively, treated and non-treated wood. Given the aforementioned model, the estimated parameters are the thickness $h_l$ of the treated layer, along with its most important mechanical parameters (i.e. $\rho _l,E_{L,l},E_{R,l},G_{LR,l}$), starting from the knowledge on the modal frequencies observed in treated wood and the parameters found for the untreated wood. The geometry was modified by introducing a parametric cross-section in the RT plane to account for the cupping effect^41^. Furthermore, the resulting 3D model of the plate with parametric cross-section was partitioned into two layers, with thickness $h_l$ and $h-h_l$, respectively. The arching height of the plate was controlled through the value $H_t^j$, which was monitored before and after each treatment stage. The material parameters of the untreated layer are those obtained with the Monte-Carlo simulation before the pre-treatment. The nominal values of the distribution used for the Monte-Carlo optimization are those of the untreated wood (see Table S1 in Supplementary Information). For each plate *j* and each treatment stage *t*, a dataset $\Theta \in {\mathbb {R}}^{M\times 5}$ of $M=3000$ tuples was created and fed to the FE model obtaining the first 15 modes in terms of eigenfrequency and mode shape. The ordering of the modes in the simulated data changed slightly from the reference ones due to the introduction of the arching and the variation in the material properties. Mode matching was accomplished before the optimization using the Modal Assurance Criterion (MAC) as similarity metric^42^. A *reference* set of mode shapes was obtained for each plate through simulation using the FEM model. In this case, the model was tuned to fit the FRF measured on the white specimen. For each tuple in $\Theta$, a set of *candidate* modes was synthesized and compared to the reference set. Pairs of reference and candidate mode shapes maximizing the MAC were identified at the end of the process.

An estimate based on finding the realization in the dataset that minimizes the difference between simulated and measured modal frequencies is not suitable at this stage. In fact, such a cost function exhibits too many local minima. Therefore, we extended the analysis to the lowest 200 minima. The parameters of the selected local minima are organized in histograms. Finally, the counts obtained from plates sharing the same pre-treatment were summed together in a cumulative histogram, from which the estimates have been obtained. Further details concerning the FEM modeling of the cupping phenomenon and the analysis accomplished can be found in Supplementary Information.

### SEM-EDX micro-analysis

SEM-EDX micro-analysis was performed on cross-sectional samples embedded in epoxy resin (Epofix Struers and Epofix Hardener with ratio 15:2) and dry-polished with silicon carbide fine sandpapers (1200–8000 mesh). The analyses were performed on the specimens treated with potassium hydroxide and potassium caseinate. The semi-quantitative line profiling of K was acquired from the up-radial treated surface of the specimen (including around 100 $\upmu \hbox {m}$ of the embedding resin on the top) through its depth. Measurements were acquired every 2 $\upmu \hbox {m}$ along a line, exploring around 900 $\upmu \hbox {m}$ of the cross-sectional samples. Elemental micro-analysis was carried out using the Bruker Quantax 200 (Billerica, MA, USA) energy-dispersive X-ray spectrometer coupled to the FE-SEM Tescan Mira 3XMU-series (Brno, Czech Republic) scanning electron microscope. Measurements were carried out in high-vacuum mode. This required the metallisation of the surface by coating a graphite film with the Cressington 208HR sputter coater. Spectra were collected at the following parameters: accelerating voltage equal to 20 kV, analysis time per spot equal to 100 s, and working distance equal to 15 mm. The semi-quantitative data were obtained by processing the experimental results with the EDAX Genesis software (version 6.04). An estimate of the actual penetration depths was obtained by fitting a piece-wise linear (i.e. bilinear) function to the decaying part of the profiles. Resulting depth values correspond to the location of the intersection between the two segments of the fitting function along the x-axis.

## Supplementary Information

Supplementary Information.

## Data Availability

The datasets generated and/or analysed during the current study are not publicly available due to ongoing research, but are available from the corresponding author on reasonable request.
