# Supplementary material for: The impact of alkaline treatments on elasticity in spruce tonewood
Source: Sci Rep. 2022 Aug 3;12:13335. doi: 10.1038/s41598-022-17596-z (PMC9349190; doi:10.1038/s41598-022-17596-z)
Supplement: Supplementary file 1 — Supplementary Information. [file 41598_2022_17596_MOESM1_ESM.pdf]

Supplementary information to: The Impact of Alkaline Treatments on Elasticity in Spruce Tonewood

Raffaele Malvermi[1,*], Michela Albano[2,3], Sebastian Gonzalez[1], Giacomo Fiocco[2], Fabio Antonacci[1], Marco Malagodi[2,3], and Augusto Sarti[1]

[1]Musical Acoustics Lab at the Violin Museum of Cremona, DEIB-Politecnico di Milano, Cremona Campus, Cremona, Italy
[2]Arvedi Laboratory of non-Invasive Diagnostics, CISRiC, University of Pavia, Via Bell'Aspa 3, 26100 Cremona, Italy
[3]Department of Musicology and Cultural Heritage, University of Pavia, 26100 Cremona, Italy
[*]raffaele.malvermi@polimi.it

ABSTRACT

This document contains additional information that is useful for reproducing the FEM-based optimization methodology described in the manuscript. A first section focuses on the compensation of the impact of the cupping phenomenon on the modal frequencies. The second section summarizes the Caldersmith's equations, used as initial estimate of the Young's moduli. Finally, the last section is dedicated to summarizing the input parameters to the FEM models and the final values of the material parameters for the white plates.

Cupping phenomenon: FEM modeling

One-sided applications of water-based chemicals usually cause the so-called *flying wood* or *cupping* phenomenon in thin flat plates, i.e. where the thickness is negligible if compared to the other dimensions[1]. In this case, a different moisture exchange rate characterizes the two faces of the plate during the drying process and the structure is subject to stresses along its principal axes. The consequent arching imposed to the plate results in an increase of the frequency for all its longitudinal bending modes[2], i.e. modes characterized by nodal lines along the grain, which is additional with respect to the effect of the treatment.

We implemented a Finite Element model to predict and compensate the frequency change caused by the presence of the curvature in treated plates. Let us assume the original plate in the (L × R × T) reference system with length L, width W, thickness h and a rectangular cross-section of dimensions ($W \times h$). The cross-section of the new pre-stressed structure, presenting an arching with height equal to H, is defined as the area between two concentric circular sectors defined on the RT plane (Fig. S1a). The center, radii and angle of aperture characterizing the two sectors are defined starting from the values measured for W, h and H as

$$z_0 = -\frac{(\frac{W}{2})^2 - H^2}{2H}, \tag{1a}$$

$$r_{in} = \sqrt{\left(\frac{W}{2}\right)^2 + z_0^2}, \tag{1b}$$

$$r_{out} = r_{in} + h, \tag{1c}$$

$$\alpha = \arccos\left(\frac{W}{2r_{in}}\right), \tag{1d}$$

such that $(0, z_0)$ is the location of the center common to the two circumferences, r_{in} is the radius associated to the downward face of the shell, r_{out} is the radius of its upward surface and α is the angle of the circular sector including the cross-section. The final 3D geometry is built as the extrusion of the parametric cross-section along the L-axis (Fig. S1b).

The impact of the cupping effect on the measured modal frequencies was analysed in COMSOL Multiphysics performing an eigenfrequency study with H varying in the range $[0, 5]$ mm and considering a step of 0.5 mm. Simulations were repeated for

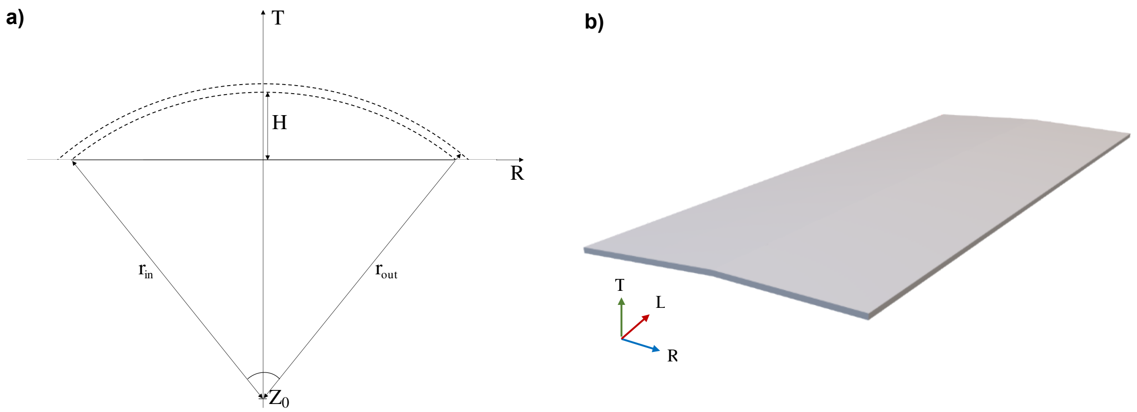

Figure S1. (**a**) Schematic drawing for the generation of the parametric cross-section on the RT plane. The center coordinate z_0 along the T-axis and the radius of the two circumferences r_{in}, r_{out} are controlled by the arching height H using Eq. (1a)-(1d). (**b**) 3D model of the arched specimen resulting from the extrusion of the cross-section along the L-axis.

each plate, setting the material properties of the FE model to the values found through the Monte-Carlo optimization presented in[3]. Results were used to compute the average variation in the frequency of the five modes of interest as a function of H, namely

$$\Delta\tilde{f}_i(H) = \frac{1}{J}\sum_{j=1}^{J}[\tilde{f}^{j}_{0,i}(H) - \tilde{f}^{j}_{0,i}(0)], \quad i = \{(0,2),(2,0),(2,1),(2,2),(2,3)\}, \tag{2}$$

where i is the mode observed and labelled using the notation based on nodal lines[4], $j \in [1,9]$ refers to the sample name, $J = 9$, $\tilde{f}^{j}_{0,i}(H)$ is the eigenfrequency simulated for mode i when plate j exhibits an arching height equal to H and $\tilde{f}^{j}_{0,i}(0)$ is the result obtained for the same mode considering the model of the original flat plate. It is noteworthy that $\tilde{f}^{j}_{0,i}(0)$ well approximates $f^{j}_{0,i}$ extracted from FRFs measured on untreated plates.

The statistics of Eq. (2), i.e. mean and standard deviation, were analysed to assess the impact of the cupping effect on each mode observed and the variability of the variation against variability in the material parameters (Fig. S2). Among the

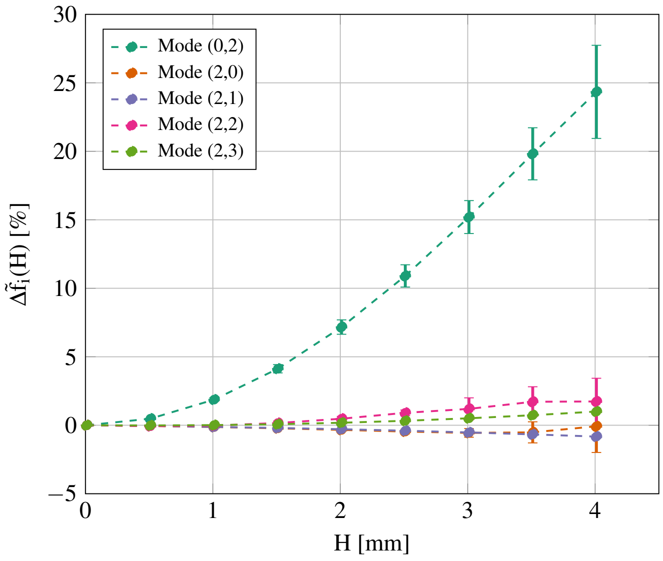

Figure S2. Averaged variation in frequency $\Delta\tilde{f}_i(H)$ computed for the first five modes observed. Mean and standard deviation were obtained varying the material parameters of the nine specimens and are shown in percentage as a function of H. The range considered for the arching height was defined accordingly to values collected from measurements.

modes, only (0,2) is strongly affected by the presence of the arching, showing a linear increase of its frequency up to 30% in the considered range of H. This was expected since the mode shape associated to mode (0,2) is characterized by the absence of nodal lines along the L direction (see Fig. 2 in the paper, inset), making the stiffness 'perceived' by the mode more sensible to changes along the width of the structure, i.e. where the bending is applied, accordingly to similar case studies in the literature[5]. The same interpretation follows for the other modes, where the relative variations show small percentages. In this case, mode shapes are characterized by the presence of nodal lines orthogonal to the plate width and thus less sensible to its bending.

It can be noticed that the variation in frequency due to the cupping phenomenon proved to be stable with respect to the variability in the material properties of the samples. Indeed, the maximum standard deviation encountered for $\Delta\tilde{f}_i(H)$ in the range of H considered is less than 5%. As a consequence, we can assume that the contribution of the material to the alteration of the modal frequencies can be decoupled from that of the arching without significant loss in accuracy.

Modal frequency estimation and initial assessment of the mechanical parameters

The frequencies of the first 5 modes observed were extracted from the measured FRFs in the range $[0,500]$ Hz, leading to

$$f^j_{t,i} \quad \text{with} \quad i = \{(0,2),(2,0),(2,1),(2,2),(2,3)\} \quad j \in [1,9] \quad t = \{0,p,s\} \text{ for } j \in [4,9] \text{ and } t = \{0,s\} \text{ for } j \in [1,3], \tag{3}$$

where subscript t refers to the actual treatment stage which the measure belongs to (0: white, p: pre-treatment, s: sizing). We employed Eq. (2) to compensate the measured modal frequencies in Eq. (3), leading to

$$\hat{f}^j_{t,i} = f^j_{t,i} - \Delta\tilde{f}_i(H^j_t), \tag{4}$$

where H^j_t is the actual value of the arching height measured for plate j at the stage t. The relative change in frequency, computed after the compensation as

$$\delta f^j_{t,i} = \frac{\hat{f}^j_{t,i} - f^j_{0,i}}{f^j_{0,i}} \quad \text{with} \quad t = \{p,s\}, \tag{5}$$

is shown in Fig. S3 and Fig. S4.

The first estimate of the Young's moduli in the longitudinal and radial directions, denoted as $\hat{E}^j_{t,L}$ and $\hat{E}^j_{t,R}$, was computed starting from the compensated frequency of modes (0,2) and (2,0) using[2]

$$\hat{E}^j_{t,L} = 12\left(\frac{2}{\pi}\right)^2\left(\frac{2}{3}\right)^4\frac{\hat{\rho}L^4\hat{f}^2_{t,(0,2)}}{h^2} \quad \text{and} \quad \hat{E}^j_{t,R} = 12\left(\frac{2}{\pi}\right)^2\left(\frac{2}{3}\right)^4\frac{\hat{\rho}W^4\hat{f}^2_{t,(2,0)}}{h^2}, \tag{6}$$

where L, W, h are the length, width and thickness of the plate, respectively, $\hat{\rho}$ is the density measured and the contribution of the Poisson's ratio is assumed to be negligible. The Young's moduli obtained from Eq. (6) are used as average values of the random distribution in the Monte Carlo procedure, as specified in the section "Methods" in the manuscript.

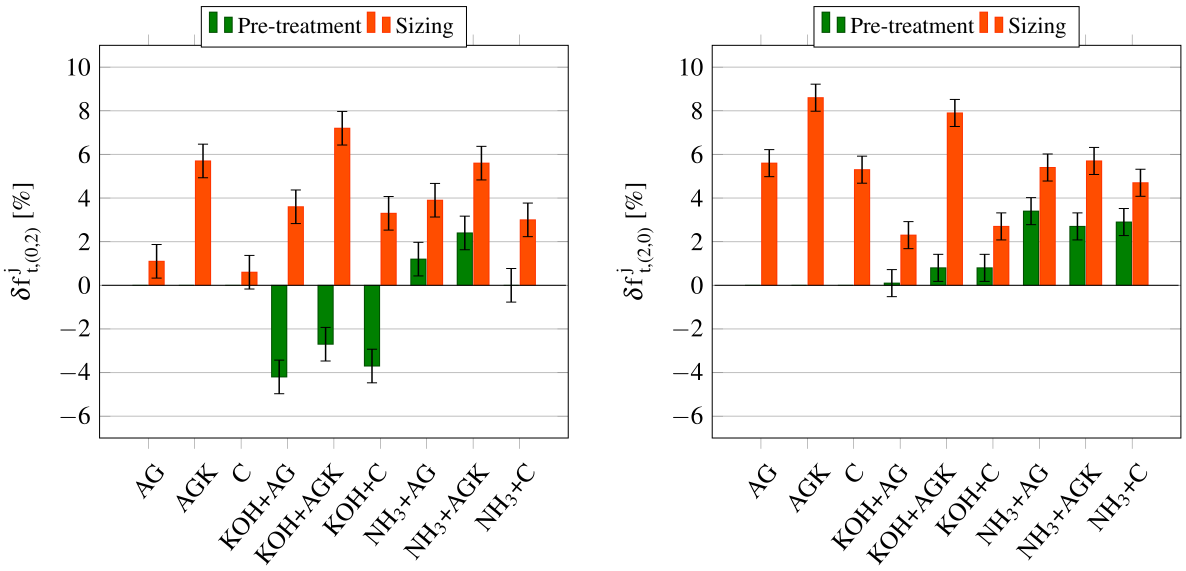

Figure S3. Relative variation in frequency $\delta f_{t,i}^{j}$ for the first bending modes, expressed in percentage and evaluated after the application each treatment stage. Specimens are ordered by primer and grouped by pre-treatment used. **Left:** First longitudinal bending mode (0,2), with maximum estimation error equal to 0.77%; **Right:** First radial bending mode (2,0), with maximum estimation error equal to 0.62%. Mode (0,2) seems more sensitive than mode (2,0) to specific combinations of treatments.

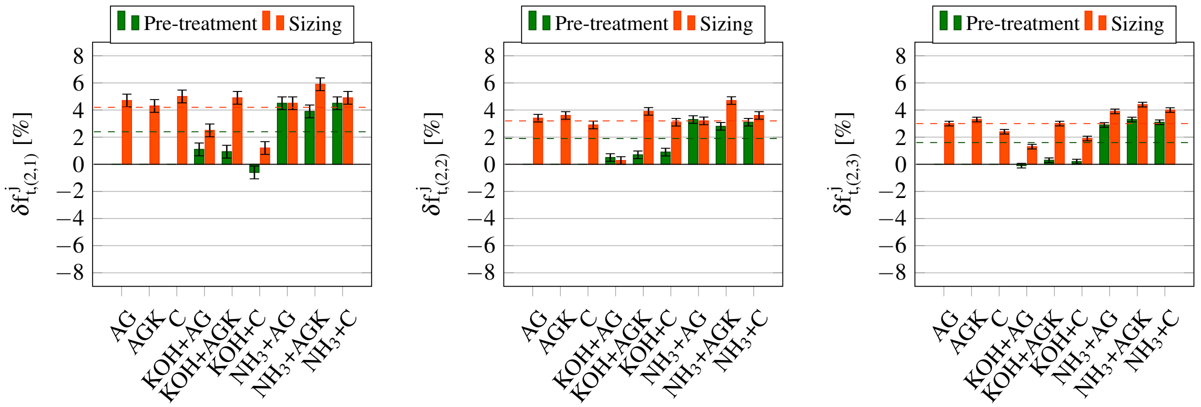

Figure S4. Frequency change $\delta f_{t,i}^{j}$ in percentage for the remaining modes. **Left:** mode (2,1) with maximum error equal to 0.47%; **Center:** longitudinal bending mode (2,2) with maximum error equal to 0.28%; **Right:** mode (2,3) with maximum error equal to 0.17%. As mode (2,0), higher modes present similar trends regardless of the primer used, with decreasing positive mean variations as frequency increases.

FEM parameters

A two-steps Monte Carlo optimization procedure is employed, supported by two Finite Element analyses. The first aims at assessing the mechanical parameters of the plate before any treatment is applied (white sample tuning). The second estimates the mechanical parameters of the treated layer (treated layer tuning). A random distribution of the mechanical parameters around a nominal value is built. The nominal value can be either taken from the literature, or a first guess is obtained from the knowledge of the modal frequencies of the plate, its geometry and density (cfr. Eq. (6)). A normal (N) or uniform (U) distribution is used, according to the specific parameter. Table S1 summarizes the FEM inputs for the two Finite Element analyses. The initial values used and the results obtained in the first step (i.e. tuning on white specimens) are reported in Table S2 for density and Young's moduli, Table S3 for the shear moduli and Table S4 for the Poisson's ratio.

**Parameter**		**Unit measure**	**Nominal value**	**FEM input (white sample tuning)**	**FEM input (treated layer tuning)**
**Plate and layer geometry**					
Plate length	L	[mm]	*from measurements*	*from measurements*	*from measurements*
Plate width	W	[mm]	*from measurements*	*from measurements*	*from measurements*
Plate thickness	h	[mm]	*from measurements*	*from measurements*	$h - h_l$
Plate arching	H	[mm]	–	–	*from measurements*
Treated Layer thickness	h_l	[mm]	–	–	$\sim U(10^{-4},10^{-3})$
**Equivalent mechanical parameters of the whole plate**					
Plate density	ρ	$[kgm^{-3}]$	*from measurements*	$\sim\rho[1+N(0,0.1)]$	*from Monte-Carlo optimization*[3]
Plate Young's moduli	E_L	[GPa]	*Caldersmith's estimation*[2]	$\sim E_L[1+U(-0.3,0.3)]$	*from Monte-Carlo optimization*[3]
	E_R	[GPa]	*Caldersmith's estimation*[2]	$\sim E_R[1+U(-0.3,0.3)]$	*from Monte-Carlo optimization*[3]
	E_T	[GPa]	$0.043E_L$[6]	$\sim E_T[1+U(-0.3,0.3)]$	*from Monte-Carlo optimization*[3]
Plate Shear moduli	G_{LR}	[GPa]	$0.064E_L$[6]	$\sim G_{LR}[1+U(-0.3,0.3)]$	*from Monte-Carlo optimization*[3]
	G_{RT}	[GPa]	$0.003E_L$[6]	$\sim G_{RT}[1+U(-0.3,0.3)]$	*from Monte-Carlo optimization*[3]
	G_{LT}	[GPa]	$0.061E_L$[6]	$\sim G_{LT}[1+U(-0.3,0.3)]$	*from Monte-Carlo optimization*[3]
Plate Poisson's ratios	μ_{LR}	–	0.372[6]	$\sim \mu_{LR}[1+U(-0.3,0.3)]$	*from Monte-Carlo optimization*[3]
	μ_{RT}	–	0.435[6]	$\sim \mu_{RT}[1+U(-0.3,0.3)]$	*from Monte-Carlo optimization*[3]
	μ_{LT}	–	0.467[6]	$\sim \mu_{LT}[1+U(-0.3,0.3)]$	*from Monte-Carlo optimization*[3]
**Layerwise mechanical parameters**					
Layer density	ρ_l	$[kgm^{-3}]$	–	–	$\sim\rho[1+N(0,0.3)]$
Layer Young's moduli	$E_{L,l}$	[GPa]	–	–	$\sim E_L[1+U(-0.3,0.3)]$
	$E_{R,l}$	[GPa]	–	–	$\sim E_R[1+U(-0.3,0.3)]$
	$E_{T,l}$	[GPa]	–	–	E_T
Layer Shear moduli	$G_{LR,l}$	[GPa]	–	–	$\sim G_{LR}[1+U(-0.3,0.3)]$
	$G_{RT,l}$	[GPa]	–	–	G_{RT}
	$G_{LT,l}$	[GPa]	–	–	G_{LT}
Layer Poisson's ratios	$\mu_{LR,l}$	–	–	–	μ_{LR}
	$\mu_{RT,l}$	–	–	–	μ_{RT}
	$\mu_{LT,l}$	–	–	–	μ_{LT}
**Rayleigh damping**					
Mass coefficient	α	$[s^{-1}]$	10	10	10
Stiffness coefficient	β	[s]	2×10^{-6}	2×10^{-6}	2×10^{-6}

Table S1. List of parameters fed to the FE model during the two-step optimization procedure. The plate dimensions and the arching due to the cupping effect were measured for all the plates before and after each treatment stage. The nominal values of the density and the Young's moduli E_L and E_R were first obtained from measurements and Caldersmith's formula[2] and then tuned through Monte-Carlo optimization to match the first modes inside the FRFs. The remaining nominal values were taken from[6] for Sitka Spruce and then optimized through Monte-Carlo optimization.

Sample	ρ [kg/m^3]		E_L [GPa]		E_R [GPa]		E_T [GPa]	
	**initial**	**optimized**	**initial**	**optimized**	**initial**	**optimized**	**initial**	**optimized**
1	353.85	349.07	11.13	10.26	1.01	0.87	0.48	0.55
2	356.25	349.30	11.05	9.69	1.06	1.03	0.48	0.42
3	411.70	396.11	14.06	11.19	0.87	0.76	0.60	0.62
4	413.01	415.41	14.13	13.10	0.79	1.04	0.61	0.67
5	416.87	407.11	14.49	11.77	0.90	1.12	0.62	0.63
6	414.62	407.02	14.43	13.41	1.03	0.95	0.62	0.55
7	436.59	438.02	14.95	14.17	0.59	0.91	0.64	0.59
8	438.42	437.97	15.78	14.43	0.72	0.91	0.68	0.65
9	387.22	385.28	10.20	9.03	0.50	0.57	0.44	0.50

Table S2. Initial and final values of the first step of Monte-Carlo optimization, i.e. the tuning of the white specimens, for the density and the Young's modulus along the three principal axis of wood.

Sample	G_{LR} [GPa]		G_{RT} [GPa]		G_{LT} [GPa]	
	**initial**	**optimized**	**initial**	**optimized**	**initial**	**optimized**
1	0.71	0.53	0.03	0.02	0.68	0.63
2	0.71	0.56	0.03	0.02	0.67	0.77
3	0.90	0.85	0.04	0.03	0.86	0.78
4	0.90	0.74	0.04	0.03	0.86	0.83
5	0.93	0.63	0.04	0.05	0.88	0.65
6	0.92	0.62	0.04	0.05	0.88	0.96
7	0.96	1.30	0.04	0.04	0.91	0.84
8	1.01	0.88	0.05	0.05	0.96	0.84
9	0.65	0.78	0.03	0.02	0.62	0.61

Table S3. Initial and final values of the first step of Monte-Carlo optimization, i.e. the tuning of the white specimens, for the shear modulus along the three principal axis of wood.

Sample	μ_{LR} [GPa]		μ_{RT} [GPa]		μ_{LT} [GPa]	
	**initial**	**optimized**	**initial**	**optimized**	**initial**	**optimized**
1	0.372	0.321	0.435	0.319	0.467	0.328
2	0.372	0.364	0.435	0.326	0.467	0.587
3	0.372	0.267	0.435	0.523	0.467	0.355
4	0.372	0.422	0.435	0.512	0.467	0.517
5	0.372	0.435	0.435	0.353	0.467	0.499
6	0.372	0.278	0.435	0.505	0.467	0.506
7	0.372	0.418	0.435	0.389	0.467	0.395
8	0.372	0.421	0.435	0.448	0.467	0.417
9	0.372	0.421	0.435	0.335	0.467	0.599

Table S4. Initial and final values of the first step of Monte-Carlo optimization, i.e. the tuning of the white specimens, for the Poisson's ratio along the three principal axis of wood.

References

1. Brandao, A. & Perré, P. The "flying wood" - a quick test to characterise the drying behaviour of tropical woods. In *5th international IUFRO wood drying conference, Québec*, 315–324 (1996).
2. Caldersmith, G. & Freeman, E. Wood properties from sample plate measurements i. *J. Catgut Acoust.* **1**, 8–12 (1990).

3. Brauchler, A., Ziegler, P. & Eberhard, P. An entirely reverse-engineered finite element model of a classical guitar in comparison with experimental data. *The J. Acoust. Soc. Am.* **149**, 4450–4462 (2021).

4. Fletcher, N. H. & Rossing, T. D. *The physics of musical instruments* (Springer Science & Business Media, 2012).

5. Emmerling, F. A. & Kloss, R. A. Freie schwingungen von flachen zylinderschalen. *Forschung im Ingenieurwesen A* **45**, 182–188 (1979).

6. Ross, R. J. *et al.* Wood handbook: wood as an engineering material. *USDA For. Serv. For. Prod. Lab. Gen. Tech. Rep. FPL-GTR-190, 2010: 509 p. 1 v.* **190** (2010).
